# Pseudarthrose de l'extrémité inférieure du fémur traitée par mégaprothèse: à propos d'un cas et revue de la littérature

**DOI:** 10.11604/pamj.2013.15.149.2583

**Published:** 2013-08-27

**Authors:** Mohammed Elidrissi, Nassereddine Hammou, Mohammed Shimi, Abdelhalim Elibrahimi, Abdelmajid Elmrini

**Affiliations:** 1 Service de chirurgie ostéoarticulaire B4, CHU Hassan II, Fez, Maroc

**Keywords:** Pseudarthrose, mégaprothèse, fémur, genou, nonunion, megaprosthesis, femur, knee

## Abstract

Les pseudarthroses de l'extrémité distale du fémur sont relativement rares du fait de la qualité de la vascularisation de cette région. La prise en charge d'une telle complication pose un certain nombre de difficultés. Le traitement chirurgical fait appel à plusieurs techniques conservatrices, le traitement par prothèse peut s'avérer utile quand la perte de substance est importante chez le sujet âgé. L'objectif de ce travail est de discuter l'intérêt de la mégaprothèse du genou dans le traitement de la pseudarthrose de l'extrémité distale du fémur, à travers l’étude de l'observation d'une patiente et revue de la littérature. Il s'agit d'une patiente âgée de 62 ans qui présente une pseudarthrose de l'extrémité distale du fémur gauche. Sur le plan clinique la patiente présente des douleurs du genou gauche, avec gène fonctionnelle importante. Le score de l'IKS préopératoire était de 60. Elle a bénéficié d'un remplacement prothétique par une mégaprothèse du genou. En postopératoire la flexion du genou était à 90°, le score de l'IKS était de 130. A travers l’étude de cette observation, et la revue de la littérature, nous pensons que l'utilisation de mégaprothèse du genou, constitue une solution efficace et durable pour le traitement des pseudarthroses du fémur distal et particulièrement chez le sujet âgé. Cette technique permet de répondre aux impératifs d'un tel aléa de la consolidation: lutter contre la douleur et garantir une mobilité satisfaisante permettant de répondre aux besoins de la vie quotidienne du patient et ainsi améliorer sa qualité de vie.

## Introduction

Les fractures de l'extrémité inférieure du fémur sont considérées comme des fractures difficiles, et ce depuis Watson-Jones qui a écrit en 1957 “peu de traumatismes présentent tant de difficultés que les fractures supracondyliennes du fémur” [1, 2]. Cependant la majorité de ces fractures peuvent être traitées chirurgicalement avec succès. Ce traitement chirurgical a comme objectif la mobilisation précoce, permettant une fonction meilleure du genou, mais parfois la pseudarthrose ou l′échec de la fixation initiale peuvent survenir, et en particulier chez les patients dont la qualité de l'os est médiocre, ou pour certains types de fractures [3–5]. Certain facteurs de risque liés au patient favorisent la survenue d'une pseudarthrose notamment l’âge avancé, l'ostéoporose, et la comorbidité [6]. Cette pseudarthrose peut survenir rarement, avec une fréquence de 0 à 6% identique quel que soit le type de traitement adopté [7]. La prise en charge d'une telle complication pose un certain nombre de difficultés, du faite de la petite taille des fragments, la perte de substance osseuse éventuelle, la présence de troubles de rotation, d'une angulation, ou d'un raccourcissement. Ces problèmes sont souvent majorés par une limitation des amplitudes articulaires du genou [2]. Le traitement chirurgical fait appel à plusieurs techniques conservatrices, mais quand la perte de substance est importante seul un traitement radical par prothèse peut s'avérer utile. L'objectif de ce travail est de discuter l'intérêt de la mégaprothèse du genou dans le traitement de la pseudarthrose de l'extrémité distale du fémur, à travers l’étude de l'observation d'une patiente et revue de la littérature.

## Patient et observation

**Figure 1:**
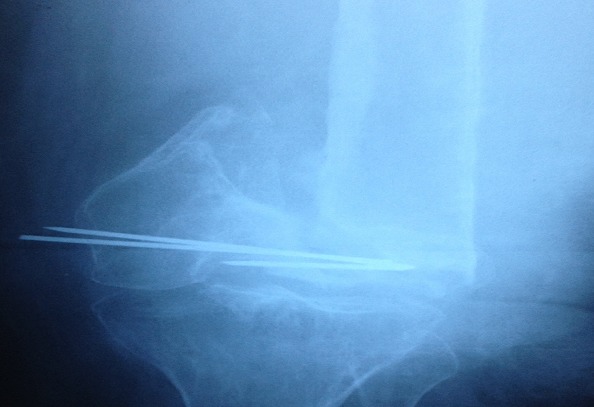
Radiographie du genou de face montrant une pseudarthrose de l'extrémité distale du fémur gauche avec une disparition quasi-totale du condyle fémoral externe

Il s'agit d'une patiente âgée de 62 ans, qui a été victime deux ans avant son admission dans notre formation d'un accident de la voie publique (collision entre deux voiture, la patiente était passagère derrière) avec point d'impact sur les deux genoux. Le bilan lésionnel initial avait objectivé une fracture sus et intercondylienne des deux fémurs traitées dans un autre établissement par plaque anatomique de l'extrémité distale du fémur de chaque côté. L’évolution a été marquée par la consolidation du côté droit, mais du côté gauche une pseudarthrose a grevé l’évolution (Figure 1). Sur le plan clinique la patiente présente des douleurs du genou gauche. A l'examen physique la marche est quasi impossible mais du côté gauche une pseudarthrose a grevé l’évolution (Figure 1).

Sur le plan clinique la patiente présente des douleurs du genou gauche. A l'examen physique la marche est quasi impossible, la patiente déambule en chaise roulante avec un raccourcissement du membre inférieur gauche de 3 cm par rapport au côté droit, l'extension a été mesurée à 0° et la flexion à 20. Le score de l'IKS (International Knee Society) en préopératoire était de 60 (score genou: 35’> l'extension a été mesurée à 0° et la flexion à 20. Le score de l'IKS (international knee society) en préopératoire était de 60 (score genou: 35, score fonction: 25). L'analyse radiographique montre une perte de substance importante notamment du condyle fémoral latéral (Figure 1). Devant l'ostéoporose importante, la perte de substance osseuse, et l’âge avancé de notre patiente un traitement conservateur parait illusoire, donc nous avons choisi de traiter la patiente par une mégaprothèse du genou.

**Figure 2:**
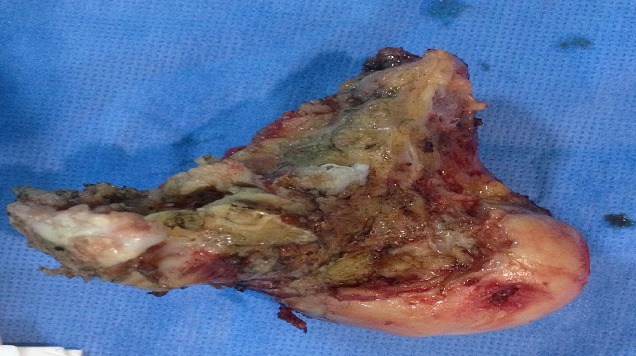
Après résection la perte de substance osseuse est importante

**Figure 3:**
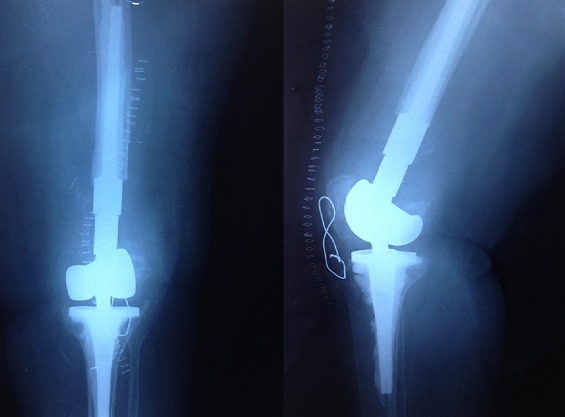
Radiographie de contrôle après mise en place d'une mégaprothèse du genou

La voie d'abord utilisée et une voie d'bord para-patellaire médiale, après extraction des broches, l'exploration peropératoire a objectivé une nécrose totale du condyle externe, avec une ostéophytose du condyle interne (Figure 2). La résection osseuse est faite en se référant à la planification préopératoire, ce qui permet de celer la tige fémorale en zone corticale de qualité normal. La langueur totale des pièces prothétique était de 12 cm (Figure 3). En postopératoire nous avons noté une nette amélioration de la douleur avec une flexion postopératoire immédiate à 50°. A un recule de 6 mois la patiente a atteint une flexion du genou à 90°, une marche avec une seule canne est devenue possible le score de l'IKS était de 130, la patiente était très satisfaite.

## Discussion

Les fractures de l'extrémité distale du fémur sont relativement fréquentes. Elles surviennent souvent suite à un traumatisme de haute énergie chez le sujet jeune, ou à basse énergie chez le sujet âgé ostéoporotique. L'objectif de toute attitude thérapeutique devant ces lésions traumatiques est de permettre une réduction anatomique du fait de leur caractère articulaire, mais également un montage stable permettant une rééducation la plus précoce possible. Le traitement chirurgical par réduction direct est le choix thérapeutique le plus souvent proposé, car c'est lui seul qui peut répondre à ces impératifs [8–10]. Les pseudarthroses en cette zone sont relativement rares du fait de la qualité de la vascularisation de cette région. Elles peuvent survenir indépendamment du traitement entretenu et à des fréquences similaires. Les séries de traitement orthopédique rapportent un taux de pseudarthrose de 2%-6% [11, 12] Ce taux est similaire à celui dans les séries du traitement chirurgical 0% - 6% [13–16]. Plusieurs facteurs favorisent la survenue d'une pseudarthrose en particulier une fixation insuffisante, la qualité médiocre de l'os, l'infection et une vascularisation médiocre qui peut être liée selon Lynch et al. [17] à une ouverture initiale, un déperiostage excessif, ou la persistance d'un defect osseux. Malgré qu'elles soient rares, les pseudarthroses de l'extrémité distale du fémur, posent un problème fonctionnel pour le patient, et un défi thérapeutique pour le chirurgien. Le traitement chirurgical conservateur a pour objectif de restaurer la continuité de l'os tout en préservant la vascularisation. Pour cela plusieurs techniques sont proposées dont la plus utilisée est l'ostéosynthèse par plaque anatomique associée à une greffe spongieuse [10]. Chez le sujet âgé, avec un os de mauvaise qualité, et une raideur articulaire très importante, et éventuellement une gonarthrose, ce traitement conservateur peut s'avérer impossible, le remplacement prothétique est alors le seul recours possible [18–20]. La prothèse massive du genou a été conçue initialement pour la reconstruction après chirurgie carcinologique, elle comporte une tige un corps et des composants articulaires [5]. Anderson a rapporté les résultats du traitement de 10 patients présentant une pseudarthrose articulaire du genou dont sept concernaient l'extrémité distale du fémur traitées par prothèse à tige longue, avec des résultats satisfaisants [21]. Haidukewych et al. [22] avaient rapporté des résultats très encourageants chez 17 patients avec un recul moyen de 5 ans.

Sur le plan technique; la présence de cicatrice antérieur, la raideur articulaire, la présence matériel d'ostéosynthèse antérieure souvent rompu, la perte de substance osseuse constituent autant de défis pour la réussite d'une telle chirurgie [22]. De ce fait la technique chirurgical passe d'abord par une bonne planification préopératoire, visant à déterminer le niveau de résection, puis le choix de la meilleure voie d'abord pour l'ablation du matériel tout en préservant les parties molles, mais également par la restauration de l'interligne articulaire ainsi que la longueur du membre [5].

A travers l’étude de cette observation, et la revue de la littérature, nous pensons que l'utilisation de mégaprothèse du genou, constitue une solution efficace et durable pour le traitement des pseudarthroses du fémur distal et particulièrement chez le sujet âgé. Cette technique permet de répondre aux impératifs d'un tel aléa de la consolidation: lutter contre la douleur et garantir une mobilité satisfaisante permettant de répondre aux besoins de la vie quotidienne du patient et ainsi améliorer sa qualité de vie. Nous ne pensons par contre pas qu'elle sera utile pour le traitement des fractures fraiches ou chez le sujet jeune, le traitement conservateur doit être tenté, et peut être utile.

## Conclusion

Les pseudarthroses de l'extrémité distale du fémur est un aléa de la consolidation rare. La mégaprothèse du genou conçue initialement pour le sauvetage du membre dans la pathologie tumorale, constitue une alternative pour le traitement chirurgical de cette entité pathologique chez le sujet âgé ostéoporotique avec une vascularisation médiocre.
